# Tricarboxylates Induce Defense Priming Against Bacteria in *Arabidopsis thaliana*

**DOI:** 10.3389/fpls.2018.01221

**Published:** 2018-08-20

**Authors:** Andrea Balmer, Victoria Pastor, Gaetan Glauser, Brigitte Mauch-Mani

**Affiliations:** ^1^Laboratory of Molecular and Cell Biology, Institute of Biology, University of Neuchâtel, Neuchâtel, Switzerland; ^2^Metabolic Integration and Cell Signaling Group, Plant Physiology Section, Department of CAMN, Universitat Jaume I, Castellon, Spain; ^3^Neuchâtel Platform of Analytical Chemistry, University of Neuchâtel, Neuchâtel, Switzerland

**Keywords:** priming, TCA, induced resistance, citrate, fumarate, primary metabolism, carboxylic acids

## Abstract

Exposure of plants to biotic stress results in an effective induction of numerous defense mechanisms that involve a vast redistribution within both primary and secondary metabolisms. For instance, an alteration of tricarboxylic acid (TCA) levels can accompany the increase of plant resistance stimulated by various synthetic and natural inducers. Moreover, components of the TCA flux may play a role during the set-up of plant defenses. In this study, we show that citrate and fumarate, two major components of the TCA cycle, are able to induce priming in Arabidopsis against the bacterial pathogen *Pseudomonas syringae* pv. *tomato* DC3000. Both citrate and fumarate show no direct antimicrobial effect and therefore enhanced bacterial resistance found *in planta* is solely based on the induction of the plant defense system. During the priming phase, both TCA intermediates did not induce any changes in transcript abundances of a set of defense genes, and in phytohormones and camalexin levels. However, at early time points of bacterial challenge, citrate induced a stronger salicylic acid and camalexin accumulation followed later by a boost of the jasmonic acid pathway. On the other hand, adaptations of hormonal pathways in fumarate-treated plants were more complex. While jasmonic acid was not induced, mutants impaired in jasmonic acid perception failed to mount a proper priming response induced by fumarate. Our results suggest that changes in carboxylic acid abundances can enhance Arabidopsis defense through complex signaling pathways. This highlights a promising feature of TCAs as novel defense priming agents and calls for further exploration in other pathosystems and stress situations.

## Introduction

The continuous exposure of plants to biotic stressors and environmental changes forces them to constantly remodel their defense strategies as well as their metabolism (Stael et al., 2011; Nomura et al., 2012; Zhang et al., 2014b; Van Aken et al., 2016). Hence, there is a massive reprogramming of the plant cell in order to activate and deploy an efficient immune reaction in response to different kinds of stress. In this context, the phenomenon of priming has been well described to be part of the resulting intricate networks of inducible defenses. In this regard, priming is defined as an induced state whereby basal and further layers of defense are potentiated to react more rapidly and more efficiently to a stress (Conrath et al., 2002; Pastor et al., 2013). The induction of these defense mechanisms involves the regulation of defense gene expression, the release of plant hormones like auxin, abscisic acid (ABA), jasmonic acid (JA), salicylic acid (SA) and/or the induction of secondary metabolites (reviewed by Balmer et al., 2015) as well as a massive redistribution of energy (Bolton, 2009; reviewed by Wagner et al., 2018). During the priming phase induced by β-aminobutyric acid (BABA), a series of metabolic changes occur which are generally characterized by a massive boost of the primary metabolism through a specific accumulation of tricarboxylic acids (TCAs) such as citrate, fumarate, (S)-malate and 2-oxoglutarate (Pastor et al., 2014). Hence, it is possible that BABA-induced priming functions through the potentiation of the TCA flux and that the primary metabolism plays an important role in BABA-induced priming.

The TCA flux could occur in a cyclic or no-cyclic fashion depending on the physiological and metabolic demands of the plant cells (reviewed by Sweetlove et al., 2010) and is a central pathway for the generation of primary metabolites. Especially in terms of energy metabolism, it is commonly thought of being important during aerobic processes and also responsible for oxidation of a major part of carbohydrate, fatty acid, amino acid and respiratory substrates to drive ATP synthesis and energy production (Fernie et al., 2004). Significant research efforts have concentrated on exploring by which means plants are able to recruit and redistribute energy flows. However, the specific role of most of the primary metabolism compounds during plant defense responses is still not fully understood. The association between primary metabolism and plant defenses has been often elucidated by analyzing the expression of genes encoding transcription factors, metabolic enzymes or by metabolomic analysis (Bolton, 2009). For example, an exposure of Arabidopsis plants to biotic stresses such as a virulent pathogen (*Phytophthora infestans*), avirulent pathogens (*Pseudomonas syringae* pv. tomato *Hrc-* and *AvrRpm1*), and to pathogen-derived elicitors (flagellin and Hairpin elicitor protein) led to up-regulation of transcripts from specific functional categories associated with primary metabolic pathways such as synthesis or degradation of carbohydrates, amino acids and lipids (Rojas et al., 2014; Schwachtje et al., 2018). These findings suggest that the primary metabolism could modulate signal transduction cascades that lead to plant defense responses (Less et al., 2011). In response to biotic stress, the enhanced demand for carbon can be provided by TCA intermediates through different pathways. Starting from amino acids, the enzyme glutamate dehydrogenase (GDH) can release amino nitrogen to yield a keto-acid that can be used in the TCA flux. In fact, the 20 proteinogenic amino acids can be metabolized into some of the seven intermediates (α-ketoglutarate, acetoacetate, acetyl-CoA, fumarate, oxaloacetate, pyruvate, and succinyl-CoA), which are critical for energy generation in plants (Miflin and Habash, 2002). On the other hand, using either glutamate or α-ketoglutarate as substrates, the γ-aminobutyric acid (GABA) pathway produces succinate, a component of the TCA flux (Shelp et al., 1999). GABA is known to be involved in the resistance response to pathogens (Bolton et al., 2008). Under particular energetically demanding conditions, the GABA shunt can provide means to utilize excess of pyruvate for energy production. Moreover, during a hypersensitive response (HR), GABA induction may also provide a way of keeping NADH-generation unaltered through the TCA flux by avoiding enzymes like aconitase, succinyl-CoA ligase, and α-ketoglutarate dehydrogenase which are inactivated under oxidative stress conditions (Tretter and Adam-Vizi, 2000; Sweetlove et al., 2002). Another potential source to reply to the high energy demand during plant defense is the degradation of fatty acids during β-oxidation. This reaction takes place in the glyoxylate cycle that mediates the conversion of acetyl-CoA to succinate; the latter is transported from the glyoxysome to the mitochondria, where it can be employed in the TCA flux. This reaction has been shown to be characteristic of Arabidopsis defense responses to *Pseudomonas syringae* (Scheideler et al., 2002).

Besides supporting higher energy demand during biotic stress situations, TCA flux intermediates such as citrate are presumed to be important players in gene expression and metabolite signaling in various prokaryotic and eukaryotic organisms (McCammon et al., 2003; Wellen et al., 2009; Yang et al., 2012). Hence, tricarboxylates could act as direct defense signals. In Arabidopsis plants, manipulating levels of TCA flux intermediates induced strong changes in transcript abundances (Finkemeier et al., 2013). However, transcriptional changes caused by TCA intermediates are specific for each metabolite. For example, supplying tobacco leaves with 40 mM 2-oxoglutarate led to a strong induction of *NITRATE REDUCTASE* but only to a faint upregulation by 40 mM malate or citrate (Müller et al., 2001). Additionally, high similarity in the transcript response was observed when comparing microarray data sets of Arabidopsis plants treated with citrate with arrays of several biotic stress experiments like *Pseudomonas syringae* infection or *flg22* treatment. The behavior of specific transcript induction or repression by the intermediates of the TCA cycle confirms that these metabolites can act as signaling molecules and strongly supports a positive interconnection between TCA components and plant defenses (Finkemeier et al., 2013).

Changes in carboxylic acid levels were demonstrated to be perceived in plants during stress responses. Additionally, during priming induced by BABA, the specific induction of TCAs indicates that components of this pathway may play a role during priming mechanisms. So far it remains ambiguous if TCAs are pivotal players in the chemical orchestra of BABA-induced priming, or whether TCAs could act alone as novel priming signals. Hence, the present study aims to assess the relationship between specific TCAs and priming in order to find out if TCAs are necessary components for an accurate deployment of priming defenses. Here, the TCAs citrate and fumarate are identified as inducers of BABA-independent priming of resistance against the bacterial pathogen *Pseudomonas syringae* DC 3000. Expression patterns of selected defense genes and phytohormone levels were analyzed to identify possible defense pathways involved during TCA-induced priming and defense responses. Our results advocate adding TCA intermediates, in particular citrate and fumarate, to the chemical spectrum of plant priming inducers. A better understanding of a putative role of TCAs in priming could provide a means to enhance stress tolerance and, thus, productivity in crop species.

## Materials and Methods

### Plant Material and Growth Conditions

*Arabidopsis thaliana* genotypes *sid1-2, jin1, coi1* and Col-0 (Provided by C. Nawrath, University of Lausanne, Switzerland; J. Turner, University of East Anglia, Norwich, United Kingdom and Lehle Seeds, Round Rock, TX, United States, respectively) were germinated in 33 mL hydrated Jiffy pellets maintained at 21°C day/19°C night, with 9 h of light (120 μE m^−2^ s^−1^) and 60% of relative humidity. One week after germination seedlings were individually transferred to Jiffys and kept in the same conditions until the chemical treatments and infection assays. All biological assays were performed with 4–5 weeks old plants.

### Treatment of Plants With Chemicals

All the chemicals used in this study were obtained from Sigma-Aldrich^1^. Four to five week-old Col-0 plants were soil-drenched with a final concentration of the carboxylic acids citrate (Trisodium citrate dihydrate, reference No. *S 1804*) and fumarate (Fumaric acid, reference No. *A 47910*) at 0.1, 1, 5, 10 mM, hydrochloric acid (Hydrochloric acid solution, reference No. *H9892*; HCl at pH 2.5 as control), 250 μM of BABA (DL-3-Aminobutyric acid, reference No. *A44207*, racemate) or water (control), were applied as soil drench 2 days prior to inoculation with bacteria (Slaughter et al., 2011). Samples were taken at 48 h post treatment (hpt). Concentrations of exogenous TCAs treatments were selected beforehand based on the fact that citrate is one of the most abundant carboxylic acids in plant cells with concentrations of about 1–5 mM in the cytosol (Martinoia and Rentsch, 1994).

### Pathogen Cultivation and Inoculation

The virulent bacterial strain *Pseudomonas syringae* pv tomato DC3000 (*Pst*DC3000) was grown overnight in liquid King’s medium B (King et al., 1954) amended with the antibiotic rifampicin (50 μg mL^−1^) for selection (Flors et al., 2008). Plants were inoculated 2 days after the chemical treatments by dipping the leaves in a *Pst*DC3000 suspension containing 10^6^ colony-forming units (cfu) mL^−1^ in 10 mM MgSO_4_ and 0.001% v/v Silwet L-77^2^ for 4 s. Mock-treated plants were dipped in the same solution without bacteria. Samples were taken at 6, 24, 48, and 72 h post infection (hpi).

### Monitoring of Disease Symptoms and Direct Toxic Effects

The disease phenotype was assessed 24, 48, and 72 hpi by counting the cfu of bacteria per gram of fresh material using the serial dilution method as described by Flors et al. (2008), or at 72 hpi by calculating the percentage of symptomatic leaves. To test the effect of TCAs on bacterial growth, King’s medium B was enriched with TCAs (citrate and fumarate, respectively) at 0.1, 1, 5, 10 mM final concentration, control medium containing rifampicin (50 μg mL^−1^) and sterile water. To maintain a stable pH, the culture medium was buffered by adding MES. Twenty μL from the stock culture of *Pst*DC3000 (2 × 10^9^cfu mL^−1^) were added to each replicate (12 mL tube), and inoculated tubes were incubated overnight in a horizontal shaker at 28°C. Subsequently, *Pst*DC3000 growth was measured by optical density at 600 nm (OD_600_).

### TCAs Measurement

Plant samples were powdered and freeze dried for TCA quantification. Five milligrams of powdered dry material were transferred into a 1.5 mL microcentrifuge tube. Samples were hydrated and homogenized with 1 mL of HCOOH 0.1% and the extraction was performed twice, with a final volume of 2 mL. Sample homogenization and hydration were made with a solution of HCOOH 0.1% containing a mix of internal standards ^13^C_6_ –citric acid, ^13^C_4_ –succinic acid and ^13^C_4_ –fumaric acid that were added during the first extraction with 100 μg L^−1^ as a final concentration. The extraction was performed introducing glass beads (2 mm Ø) into each tube and using a mixer mill at a frequency of 30 Hz during 3 min. Tubes were centrifuged at 14000 rpm at 10°C and the supernatant was recovered and placed into a new tube. The two extractions were joined and filtered through a 0.2 μm cellulose acetate filter. An aliquot of 20 μl was injected into the LC-MS/MS instrument, an Acquity Ultraperformance Liquid Chromatography system connected to a triple quadrupole mass spectrometer (UPLC-TQD, Waters, Manchester, United Kingdom). The separation of the TCAs was performed with a column (Acquity UPLC HSS T3 2.1 × 100 mm, 1.8 μm) maintained at 40°C. A gradient of methanol and water containing 0.1% HCOOH was used for analyte elution. The gradient elution started with a flow of 0.3 mL min^−1^ and kept in isocratic conditions during 4 min at 95% aqueous mobile solvent, that reached 60% during 2 min and left to recover for one more minute at initial conditions. The column was allowed to equilibrate for 1 min, giving 8 min per sample. Ion detection was set in negative electrospray ionization (ESI) applying 3.3 kV capillarity voltage, and using multiple reaction monitoring (MRM) mode. Drying, nebulizing and cone gas was nitrogen and for the collision gas Ar (Praxair, Valencia, Spain) was used. The desolvation gas was set at 800 and 60 L/h for the cone gas flow. Temperatures were fixed at 350°C for desolvation and 120°C for the source. The cone and collision energies were 15 V and 10 eV for all analytes. The transitions selected for citric, fumaric, malic, succinic, and 2-oxoglutaric acid (Sigma) were *m/z* 191 > 111, 115 > 71, 133 > 115, 117 > 73, 145 > 101, and for the internal standards, ^13^C_4_- fumaric acid *m/z* 119 > 74; ^13^C_4_- succinic acid *m/z* 121 > 76 and ^13^C_6_- citric acid *m/z* 197 > 116 (Cambridge Isotope Laboratories, Inc). Analyte quantification was achieved by internal calibration. For malic acid quantification, ^13^C_4_- succinic acid was used as internal standard and ^13^C_4_- fumaric acid for 2-oxoglutaric acid. The LOQ for citric, fumaric, succinic, malic acid, and 2-oxoglutaric acids were 0.31, 0.24, 0.14, 0.27, and 0.19 μg mL^−1^ correspondingly.

### Hormone Quantification

For hormone analysis, SA, JA, jasmonic acid-isoleucine (JA-Ile) and ABA were quantified simultaneously from leaf material by UHPLC-MS/MS as previously described (Glauser et al., 2014). Hormone measurements were performed in material from plants treated with chemical inducers as well as after infection with *Pst*DC3000. To analyze each condition, three independent biological replicates per sample were generated and three independent experiments were conducted.

### Camalexin Quantification

For the analysis of camalexin, fresh frozen plant material was ground to a fine powder using mortars and pestles cooled with liquid nitrogen. A 100 mg aliquot was weighed and transferred to a 1.5 mL microcentrifuge tube, to which 500 μL were added of extraction solvent (methanol:H_2_O:formic acid, 80:19.5:0.5, v/v) and 5–6 glass beads (2 mm diameter). Samples were extracted in a Retsch mixer mill for 4 min at 30 Hz, after which the extract was centrifuged and the supernatant recovered and transferred to an HPLC vial. The analysis of camalexin was performed by UHPLC-MS/MS using an Ultimate 3000 RSLC (Dionex, Thermo Fisher Scientific) coupled to a 4000 QTRAP (AB Sciex). The column was an Acquity UPLC BEH C18 (50 × 2.1 mm, 1.7 μm, Waters) maintained at 25°C and the mobile phases were H_2_O + formic acid 0.05% (phase A) and acetonitrile + formic acid 0.05% (phase B). The following gradient program was used at a flow rate of 0.4 mL/min: 5–60% phase B in 4 min, 60–100% phase B in 2 min, holding at 100% phase B for 2 min and reequilibration at 5% phase B for 3 min. The injection volume was 3.5 μL. Detection was performed in electrospray positive ionization using the MRM mode. The transition *m/z* 201/59 was used as quantifier while the transitions *m/z* 201/160, 201/142, 201/130, 201/116 and were used as qualifiers. For the quantifier transition (*m/z* 201/59), the declustering potential (DP), the collision energy (CE) and the collision exit potential (CXP) were set to 100, 51, and 10 V, respectively. Source parameters were set as follows: ion spray (IS) voltage +5.5 kV, gas temperature (TEM) 500°C, nebulizing gas (GS1), drying gas (GS2) and curtain gas (CUR) 60, 40, and 25 psi, respectively. Quantification was achieved by external calibration using camalexin^3^ concentrations at 0.2, 1, 5, 20, 100, and 500 ng/mL. The lowest limit of quantification was 0.2 ng/mL or 1 ng/g FW.

### Gene Expression Analysis

Arabidopsis plants that had been subjected to chemical treatments and inoculated with *Pst*DC3000 as described above were evaluated. RNA extraction and cDNA synthesis was performed as described before (Balmer et al., 2013). The primers used in this study and their efficiencies are listed in **Supplementary Table S1**; primer efficiency was calculated with the help of a dilution curve. Quantitative real-time PCR was performed using the SensiMix SYBR kit (Bioline)^4^ on a ROTOR GENE 6000 cycler (Qiagen). PCR reactions were conducted using three independent biological replicates per sample. PCR reactions were done in technical duplicates as a three-step reaction (initial hold step, 95°C for 10 min; 40 cycles of amplification comprising 95°C for 15 s, 60°C for 20 s and 72°C for 20 s), with a final melting curve analysis (68–95°C). Melting curve and cycle threshold analysis were performed using rotor gene 6000 software version 1.7. Gene expression of infected tissue and control plants were calculated relative to the expression of the housekeeping genes Actin and SAND using Ct delta-delta method.

### Extraction and Liquid Chromatography-Tandem Mass Spectrometry Analysis of BABA

BABA was quantified in material from plants treated with chemical inducers as well as after infection with *Pst*DC3000. Plant material was harvested, flash-frozen and ground to fine powder in liquid nitrogen. The extraction protocol was conducted as described by Thevenet et al. (2017); in brief 100 mg of ground tissue was extracted in 500 μL of 0.1% HCOOH/H_2_O (v/v) containing the deuterium labeled internal standard (BABA-d_3_) using a Retsch mixer mill. After centrifugation of the extract at 18400 *g* during 4 min, the supernatant was purified by solid phase extraction on an Isolute SCX-2 cartridge (1 mL, 100 mg). The eluate was concentrated to dryness in a centrifugal evaporator (Speedvac) at 35°C. Samples were finally resuspended in 300 μL (1: 3) organic mobile phase B/EtOH 80% (v/v) leading to a final concentration of internal standards of 50 ng mL^−1^. Extracted BABA was quantified using an Ultimate 3000 RSLC (Dionex, Thermo Fisher Scientific) interfaced with a 4000 QTRAP (AB Sciex) by injecting 3.5 μL of extract on an Acquity UPLC BEH HILIC column (100 mm × 2.1 mm, 1.7 μm, Waters).

### Statistical Analysis

Variances of quantified levels of bacterial growth, transcript abundance, fold induction of gene expression and phytohormones were analyzed by a *t*-test; a *P*-value < 0.05 was considered significant. All statistical analyses were performed using GraphPad Software^5^.

## Results

### Citrate and Fumarate Induce Resistance in Arabidopsis Against *Pst*DC3000 in a Dose-Dependent Manner

**FIGURE 1:**
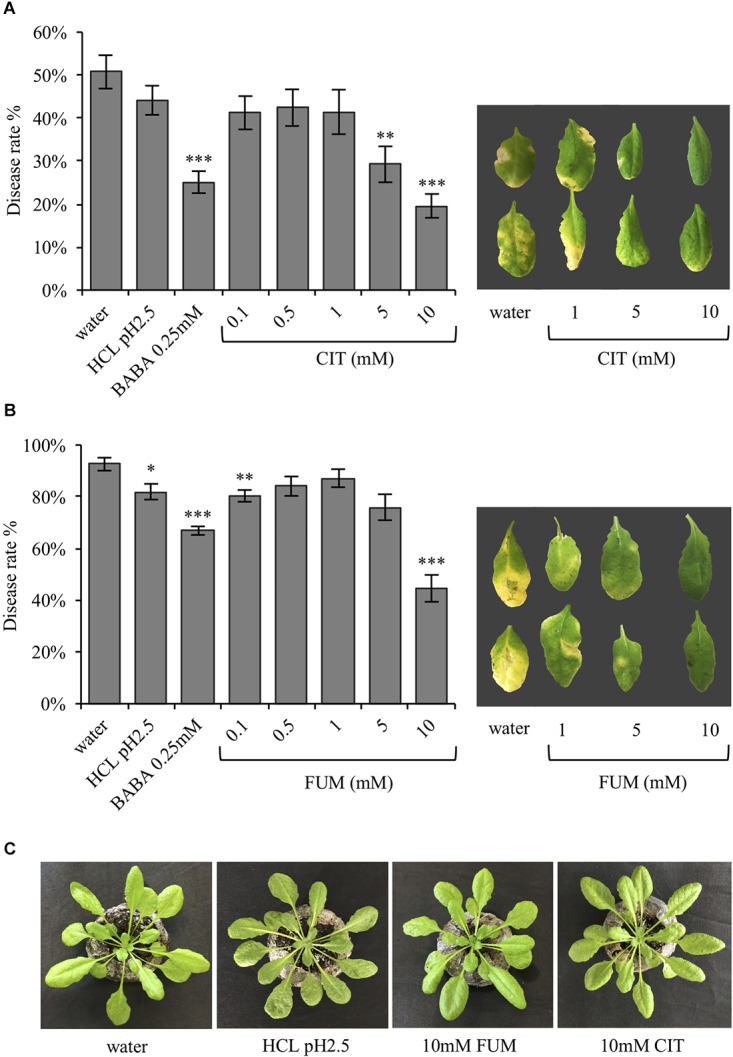
In Arabidopsis, the TCAs citrate and fumarate confer resistance to *Pst*DC3000. **(A)** Disease rate in percentage (3 dpi) of Arabidopsis plants soil drenched with different concentration of citrate, water, HCl and BABA as a controls 2 days before dip-inoculation with *Pst*DC3000 (10^6^cfu mL^−1^). Depicted leaves were collected 5 dpi. **(B)** Disease rate of Arabidopsis plants soil-drenched with various concentrations of fumaric acid, water, HCl and BABA as a controls 2 days before dip- inoculation with *Pst*DC3000 (10^6^cfu mL^−1^) is shown in percentage (3 dpi bacterial challenged). Leaves from pictured were collected 5 dpi. **(C)** Arabidopsis Col-0 plants 3 days after being soil drenched with HCl (pH 2.5), citrate 10 mM, fumarate 10 mM, and water. Data represent the mean ± SEM (*n* = 12 biological replicates). The experiments were repeated three times and a representative replicate is shown. Asterisks indicate significant differences: ^∗^*P* ≤ 0.05; ^∗∗^*P* ≤ 0.01; ^∗∗∗^*P* ≤ 0.001 (treatments vs. water) as determined by *t*-test.

It has been previously demonstrated that TCAs are upregulated during the BABA-induced priming phase, respect to the *Pst*Rpt2-induced resistance (Pastor et al., 2014). Therefore, the present study aimed to ask whether these compounds can also act as defense and/or priming inducers, as previously demonstrated for other acidic chemical signals such as azelaic acid, pipecolic acid and recently acetic acid (Jung et al., 2009; Návarová et al., 2012; Kim et al., 2017). To test the defense inducing capacity of carboxylic acids, citrate and fumarate were chosen. Their defense and priming potential was assessed in a bioassay using Arabidopsis plants treated by soil drench with both chemicals at various concentrations (0.1, 0.5, 1, 5, and 10 mM final concentration in the soil), as well as water, HCl (pH 2.5) and BABA (0.25 mM) as controls. HCl was added as a control to rule out that the observed effects were due to the low pH instead of fumarate itself. Two days after the chemical treatments the plants were inoculated with the hemibiotrophic pathogen *P. syringae* pv. *tomato DC3000.* Three days post bacterial infection the disease rate was measured as percentage of symptomatic leaves (**Figure 1**) and as cfu per gram of fresh material at 24, 48, and 72 hpi (**Supplementary Figure S1**). The treatment of Arabidopsis plants with 5 and 10 mM of citrate resulted in an induction of resistance against *Pst*DC3000 compared to water- and HCl-treated plants (**Figure 1A**). Notably, the disease rate on plants pre-treated with citrate was similar to the one observed for BABA-treated plants. Five days after inoculation, leaves from 5 and 10 mM citrate-treated plants were less affected by the bacterial infection than the corresponding controls (**Figure 1A**). Fumarate treatment at 0.1 mM also seemed to protect the plants from bacterial infection compared to water treatment. However, this difference was not statistically significant with respect to HCl treatment and could not be observed with higher concentrations between 0.5 and 5 mM (**Figure 1B**). In contrast, fumarate treatment at 10mM induced resistance to a similar extent as citrate. Importantly, HCl (pH2.5), citrate and fumarate treatments at the highest concentration (10 mM) did not affect plant growth or trigger phytotoxicity symptoms, excluding that a chemical stress induced antibacterial resistance (**Figure 1C**).

As shown in **Figure 1**, resistance induced by citrate and fumarate were dose-dependent, with 10 mM citrate and 10 mM fumarate being the most efficient concentrations for priming bacterial resistance.

### Citrate and Fumarate Have No Direct Toxicity Against *Pst*DC3000

**FIGURE 2:**
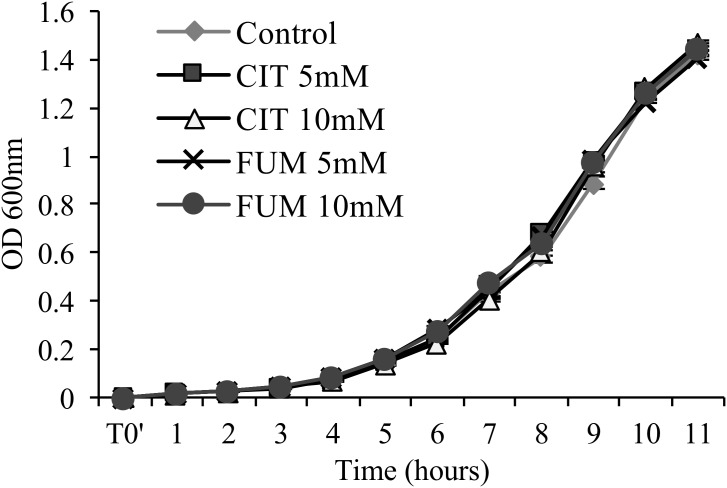
Assessment of antimicrobial activity of citrate and fumarate against *Pst*DC3000. Liquid King’s medium B was enriched with citrate and fumarate at different concentrations (5 and 10mM) and water as a control. *Pst*DC3000 growth was measured every hour during 11 h post inoculation by optical density at 600 nm. Data represent the mean ± SEM (*n* = 6 biological replicates).

To determine whether carboxylic acid compounds display direct antimicrobial activity, we performed an *in vitro* growth assay, cultivating *Pst*DC3000 in liquid King’s medium B amended with different concentrations of citrate and fumarate. Neither of the treatments reduced bacterial growth (**Figure 2**) compared to the control. As a consequence, enhanced bacterial resistance found *in planta* is solely based on the induction of the plant defense system, and not triggered by secondary antimicrobial effects of TCAs.

### TCAs Induce Defenses Independently of Endogenous BABA

**FIGURE 3:**
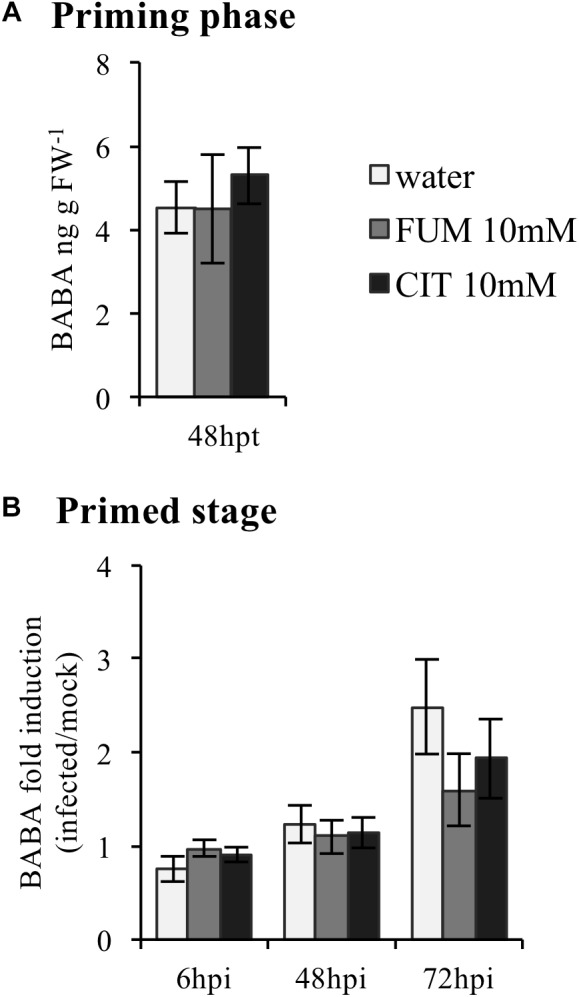
BABA induction upon treatment with citrate and fumarate in the priming phase and the primed stage. **(A)** Arabidopsis leaves were collected 48 h after soil drench with citrate and fumarate or water as a control. BABA levels are expressed in ng gFW^−1^. **(B)** BABA fold induction (infected/mock) were analyzed in plants pretreated with citrate 10 mM, fumarate 10 mM or water as a control 2 days before dip-inoculation with *Pst*DC3000 (10^6^cfu mL^−1^). Data represent the mean ± SEM (*n* = 6 biological replicates). A *t*-test was applied (water vs. treatments).

Endogenous levels of BABA have been recently demonstrated to be specifically induced by the plant’s immune system under particular biotic stress conditions such as *Pst*DC3000 infection, suggesting an involvement of endogenous BABA in plant defense responses (Baccelli et al., 2017). Furthermore, synthetic BABA treatment has been shown to prime for enhanced plant resistance, concomitantly with a boost of TCA levels (Zimmerli et al., 2001; Pastor et al., 2014). In order to analyze whether TCAs could be linked to the induction of endogenous BABA, the levels of BABA were analyzed in plants pre-treated with citrate and fumarate, both at 10 mM. Leaf material was collected 2 days post chemical treatment (priming phase) and 6, 48, and 72 h post- *Pst*DC3000 infections (the primed state for BABA measurements). During the priming phase, neither citrate nor fumarate resulted in any difference in the levels of BABA compared to water-treated plants (**Figure 3A**). Furthermore, BABA levels gradually increased during the time course of infection (**Figure 3B**). However, no difference of BABA induction was detected when comparing citrate and fumarate treatments to the water-treated controls.

### TCA Levels During the Priming Phase Induced by Citrate and Fumarate Treatments

**FIGURE 4:**
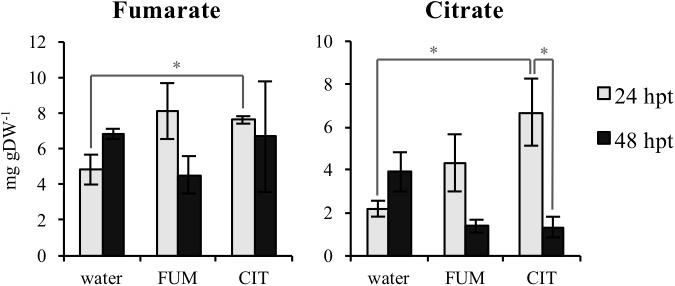
Tricarboxylic acids (TCAs) levels upon citrate and fumarate treatment during the priming phase (24 and 48hpt). Arabidopsis leaves were collected 24 and 48h after soil drench with 10mM citrate and 10mM fumarate or water as a control. TCAs levels are expressed in mg gDW^−1^. Data represent the mean ± SEM (*n* = 3 biological replicates) of one from three independent experiments. Asterisks indicate significant differences (treatments vs. control or 24 hpt vs. 48 hpt) as determined by *t*-test: ^∗^*P* ≤ 0.05.

In order to test if exogenous fumarate and citrate treatments stimulate changes in TCA intermediate composition in leaves of treated plants, concentrations of fumarate, citrate, 2-oxoglutarate, malate and succinate were measured 24 and 48 h post treatments (hpt). The time points for these measurements were selected in order to analyze the TCA levels in leaves before inoculation with the bacteria (48 hpt citrate and fumarate treatments). As shown in **Figure 4** and **Supplementary Figure S2**, 10 mM fumarate treatment did not trigger any changes in TCA intermediates at 24 hpt. At 48 hpt, fumarate treatment led to a slight reduction of malate levels compared to the water treatment (**Supplementary Figure S2**). On the other hand, exogenous citrate supplied by root drench only led to an increase of fumarate and citrate levels at 24 hpt while at 48 hpt no significant changes in TCA intermediates were found compared with water-treated plants. Additionally, upon citrate treatment, a depletion of 2-oxoglutarate and citrate was observed when comparing 24 hpt with 48 hpt. The similarity between the pattern of both TCAs was expected as citrate is one of the precursors of 2-oxoglutarate synthesis, therefore both citrate and 2-oxoglutarate levels are likely to be similarly tuned (Hodges, 2002).

### Defense-Related Genes Are Differentially Regulated in TCA-Treated Plants During the Distinct Priming Phases

**FIGURE 5:**
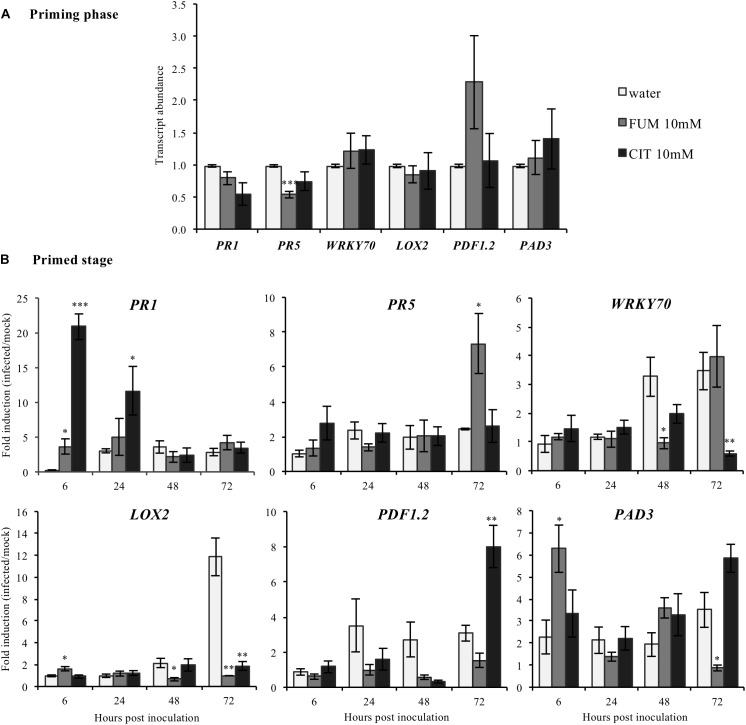
Effect of the TCAs citrate and fumarate and of *Pst*DC3000 infection on the expression of defense-related genes. **(A)** Priming phase: transcript abundance for *PR1, PR5 WRKY70*, SA signaling pathway; *LOX2* and *PDF1.2*, JA/ET biosynthesis and signaling pathway and *PAD3* camalexin biosynthesis. For the priming phase, samples consist of leaves from Arabidopsis plants soil-drenched with 10 mM citrate, 10 mM fumarate and water as a control, collected 48 h after chemical treatments. Gene expression was measured by qRT-PCR and normalized by the housekeeping genes *Actin* and *SAND*. **(B)** Primed stage: fold induction (infected/mock) of gene expression for *PR1, PR5, WRKY70*, *LOX2*, *PDF1.2*, as well as for *PAD3* were analyzed. Arabidopsis plants were soil-drenched with 10 mM citrate, 10 mM fumarate and water 2 days before dip-inoculation with *Pst*DC3000 (10^6^cfu mL^−1^). Gene expression was analyzed in a time-course of 6, 24, 48, and 72 h after infection. Data represent the mean ± SEM (*n* = 6 biological replicates). Asterisks indicate significant differences (treatments vs. control) as determined by *t*-test: ^∗^*P* ≤ 0.05; ^∗∗^*P* ≤ 0.01; ^∗∗∗^*P* ≤ 0.001.

To investigate putative transcriptional mechanisms by which citrate and fumarate may alter pathogen susceptibility in Arabidopsis, a comparative analysis of the leaf transcriptome of citrate-, fumarate- and water-treated plants under *Pst*DC3000 and mock inoculation was undertaken. Sampling was performed during the priming phase and the primed state. These two phases provide information about the mode of action of both TCAs: as inducers of resistance or/and as priming agents. Known defense genes were chosen as transcriptional markers for SA (*PR1, PR5 WRKY70*), JA (*LOX2*, *PDF1.2*) and camalexin (*PAD3*). During the priming phase, treatment with 10 mM fumarate reduced transcript abundance of *PR5* (*Pathogenesis-Related Protein5*) compared to water-treated plants (**Figure 5A**). However, the other monitored genes showed no significant changes in transcript abundance following 10mM fumarate and 10 mM citrate treatments compared to water-treated plants (**Figure 5A**).

During the priming state, however, treatment with 10 mM citrate resulted in a strong induction of *PR1* (*Pathogenesis-Related Protein1*) at early time-points (6 and 24 hpi) compared to water-treated plants while at 72 hpi, *PDF1.2* was found to be upregulated (**Figure 5B**). Interestingly, citrate-treated plants showed a significant reduction of the WRKY transcription factor 70 (*WRKY70*) at 72 hpi, and of *LOX2* which codes for the Lipoxygenase 2, (**Figure 5B**). Moreover, plants treated with 10 mM fumarate showed a different gene induction pattern compared to 10 mM citrate-treated plants. Fumarate treatment slightly increased the induction levels of *PR1, LOX2* and *PAD3* genes at early time points after *Pst*DC3000 infection. Levels of *PR5* were also slightly increased at 72 h after *Pst*DC3000 infection (**Figure 5B**). *LOX2, PAD3* and *WRKY70* were found to be down-regulated in fumarate treatments at later time points compared to water.

### Analysis of Arabidopsis Phytohormones and Camalexin During the Priming Phase and the Primed Stage Induced by Citrate and Fumarate

**FIGURE 6:**
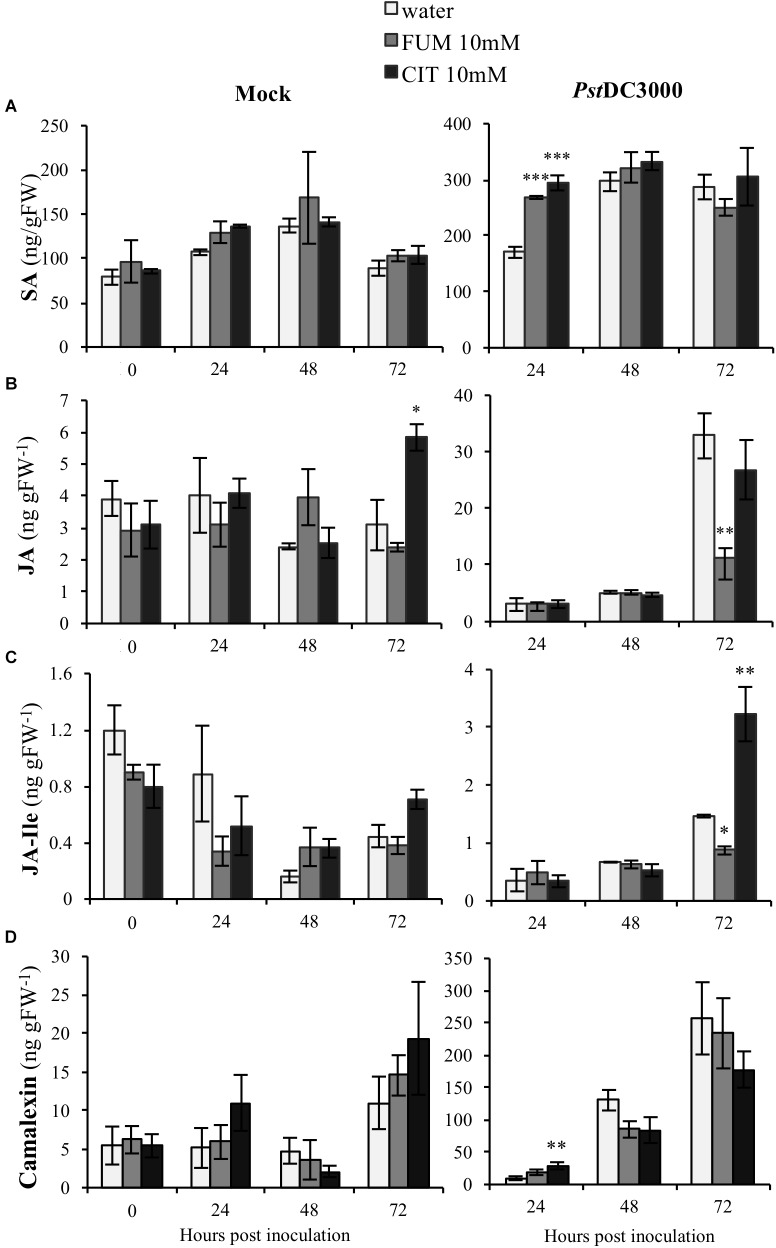
Effect of the TCAs citrate and fumarate and *Pst*DC3000 infection on the accumulation of SA, JA, JA-Ile and camalexin. **(A)** SA levels (ng gFW^−1^), **(B)** JA levels (ng gFW^−1^), **(C)** JA-Ile levels (ng gFW^−1^) and **(D)** camalexin (ng gFW^−1^) during the priming phase (0 h post inoculation), 24, 48, and 72 h post mock and *Pst*DC3000 inoculation. Priming phase samples consist of soil-drenched Arabidopsis plants with 10 mM citrate, 10 mM fumarate and water as a control. The material was collected 48 h post chemical treatments. Mock and *Pst*DC3000 samples are Arabidopsis plants soil-drenched with 10 mM citrate, 10 mM fumarate and water 2 days before dip-inoculation with *Pst*DC3000 (10^6^cfu mL^−1^). Data represent the mean ± SE (*n* = 3 biological replicates). These experiments were repeated on three independent occasions and a representative replicate is shown. The Asterisks indicate significant differences (treatments vs. control) as determined by *t*-test: ^∗^*P* ≤ 0.05; ^∗∗^*P* ≤ 0.01; ^∗∗∗^*P* ≤ 0.001.

Plant hormones are well-known to be key players in plant-induced responses by virulent *Pseudomonas* bacteria. *Pst*DC3000 interaction with Arabidopsis is mediated by interplay of both SA and JA (Brooks et al., 2005). Moreover, our results shown here demonstrate that both citrate and fumarate treatments induced specific changes at the transcriptomic level of several phytohormone-related genes. Subsequently, in order to determine if citrate and fumarate could modulate phytohormone induction during both the priming phase (**Figure 6**; 0 h post inoculation) and the primed stage (upon *Pst*DC3000 infection), we measured the levels of SA, JA and jasmonoyl-isoleucine (JA-Ile) in Arabidopsis plants after 10 mM citrate, 10Mm fumarate and water treatments and upon mock and *Pst*DC3000 infection. During the citrate- and fumarate-induced priming phase (48 h post treatments with the chemicals), SA, JA and JA-Ile levels did not exhibit any statistically significant differences compared to water-treated plants. Besides, under mock conditions, JA accumulated to a slightly higher extent in citrate- than in water-treated plants (**Figure 6B**). On the other hand, in fumarate and citrate-treated plants, *Pst*DC3000 inoculation significantly induced SA accumulation at 24 hpi. Fumarate-treatment reduced JA and JA-Ile levels at later time point after infection compared to water-treated plants (**Figures 6B,C**). Additionally, at 72 hpi, JA-Ile accumulated in citrate-treated plants infected with *Pst*DC3000. These results suggest that citrate and fumarate did not affect SA, JA or JA-Ile levels in absence of bacterial infection. However, priming by both TCAs resulted in changes in SA, JA and JA-Ile at early and later times points post *Pst*DC3000 infection. In parallel to the above described phytohormones, levels of the camalexin were also measured in the same experimental conditions. Camalexin is one of the main phytoalexins produced by Arabidopsis. Its accumulation in plants under pathogen attack is mediated by a complex phytohormone balance and inhibits the growth of virulent strains of *Pseudomonas* (Heck et al., 2003). During the priming phase induced by citrate and fumarate and under mock conditions, camalexin levels did not show any statistically significant differences compared to water-treated plants (**Figure 6D**). Notably, basal levels of camalexin in Arabidopsis plants were found to be around 0.3 ng gFW^−1^ as show in **Supplementary Figure S3**. In the experimental conditions applied in the assays above, plants from priming phase (**Figure 6D**; 0 h post inoculation) samples were exposed to 100% relative humidity and mock samples to 100% relative humidity, 10 mM MgSO4 and 0.001% Silwet. These factors could explain the augmented camalexin levels (∼10 ng gFW^−1^) measured at PP and mock time points (**Figure 6B**). Subsequently, camalexin levels gradually increased during the time course of infection under all treatments. Altogether, only citrate induced significantly camalexin at an early time point upon *Pst*DC3000 infection compared to water-treated plants (**Figure 6D**).

### Priming Induced by Citrate and Fumarate Is SA- and JA-Dependent

**FIGURE 7:**
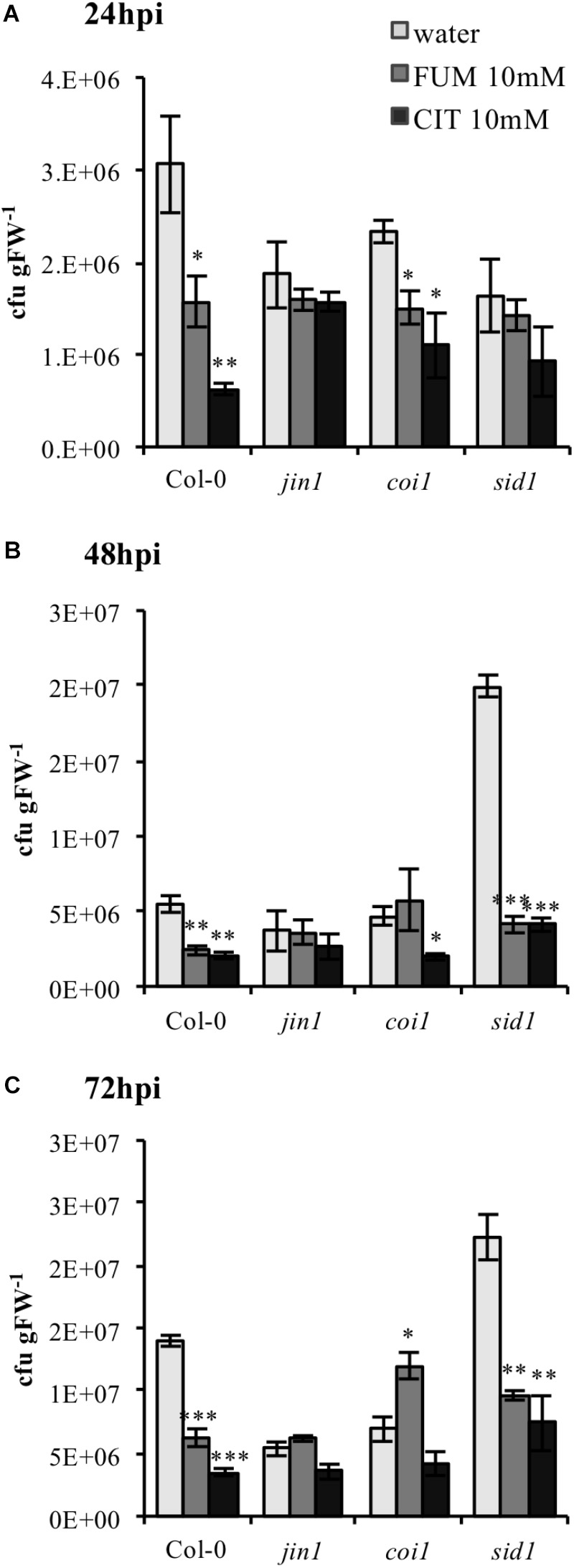
Citrate- and fumarate-induced priming against *Pst*DC3000 in Arabidopsis mutants impaired in SA and JA signaling. Arabidopsis genotypes *jin1, coi1, sid1* and the wild type Col-0 were soil drenched with 10 mM citrate, 10 mM fumarate and water as a control 2 days before dip-inoculation with *Pst*DC3000 (10^6^cfu mL^−1^). The disease rate was expressed as cfu gFW^−1^ and analyzed in a time-course of 24 h **(A)**, 48 h **(B)**, and 72 h **(C)** after infection. Data represent the mean ± SE (*n* = 3 biological replicates). This experiment was repeat three time with similar results. Asterisks indicate significant differences (treatments vs. control) as determined by *t*-test: ^∗^*P* ≤ 0.05; ^∗∗^*P* ≤ 0.01; ^∗∗∗^*P* ≤ 0.001.

Upon bacterial infection, *Pst*DC3000 virulence factors like coronatine (COR) are well known to act as manipulators of host phytohormone signaling pathways (Yi et al., 2014), demonstrating the cardinal role of plant hormones in bacterial defense. Therefore, we investigated if modifications of phytohormone synthesis and signaling were crucial in triggering the phenotype of priming induction exhibited under both treatments. A set of established Arabidopsis mutants impaired in SA and JA signaling and synthesis were selected to monitor the efficiency of TCA-induce priming. Both mutants and wild type (Col-0) plants were treated with 10 mM citrate and 10 mM fumarate and subsequently inoculated with *Pst*DC3000. To assess the importance of JA, the *jin1* mutant (*jasmonate-insensitive 1*; also known as *MYC2*), – a mutant well described to exhibit reduced susceptibility to *Pst*DC3000 compared to wild type (Lorenzo et al., 2004) was tested first. As expected, this mutant showed reduced susceptibility to *Pst*DC3000 infection upon water treatment compared to wild type Col-0. Notably, *jin1* was impaired in mounting citrate- and fumarate-induced priming against *Pst*DC3000 at all measured time points (**Figures 7A–C**). Similarly, *coi1* (*coronatine insensitive1*, a mutant which shows increased resistance to *Pst*DC3000, is jasmonate insensitive and has increased level of SA upon infection) (Thines et al., 2007), showed a slightly lowered capacity to be primed by citrate and fumarate. This was obvious at all the time points. In particular, at 72 hpi this mutant did not induce resistance upon citrate treatment and displayed a higher bacterial pathogen load in fumarate- than in water-treated plants (**Figure 7C**). Furthermore, to address the importance of the SA pathway, the *enhanced disease susceptibility5* (*eds5;* also known as *sid1*) mutant was subjected to TCA-induced priming. *eds5/sid1* is an essential component of SA-dependent signaling for disease resistance and is SA-deficient (Nawrath and Métraux, 1999). As expected, *sid1* showed high susceptibility to *Pst*DC3000 in water-treated plants at 48 and 72 hpi (**Figures 7B,C**). At early time point (24 hpi), *sid1* was unable to mount a priming response triggered by citrate or fumarate (**Figure 7A**).

## Discussion

The chemical profile during priming described in a previous study (Pastor et al., 2014) offered intriguing information about metabolic pathways that could be implicated in priming. Since a general upregulation of TCAs was observed during BABA-induced priming it was hypothesized that they might act as important players during priming responses. TCA flux intermediates such as citrate have been identified as important players in gene expression and metabolite signaling in plants Finkemeier et al., 2013). In the present study, citrate and fumarate were shown to have the potential to prime for augmented resistance against *Pst*DC3000 in Arabidopsis. Moreover, these compounds did not display *in vitro* antibacterial activity, suggesting that they exert their mode of action through the plant immune system. Until now, the role of TCAs during induced resistance has remained unclear, but recent work started to uncover direct links between TCAs and plant defense. Often, alterations of TCA levels are characteristically associated with a pathogen challenge, but it was not clear if these alterations played a direct defensive role by interfering with pathogen development, if they mirrored a metabolic phenotype during biotic stress situations, or simply reflected a physiological status of high energy demand during plant defense. Notably, an enhanced resistance to *Pst*DC3000 infection was observed in knockout lines of the cytosolic NADP-isocitrate dehydrogenase (Mhamdi et al., 2010). These plants were shown to accumulate up to 2-fold more citrate, suggesting a resistance enhancing effect of higher citrate levels. Recently, enhanced levels of TCA intermediates in the Arabidopsis mutant Chi-CWP2 (expressing a class-I chitinase and a *Fusarium*-specific recombinant antibody gene) could be linked to resistance against *Fusarium graminearum* (Chen et al., 2017). On the other hand, TCA cycle inhibition by monofluoroacetate (MFA) triggers citrate accumulation and increases the susceptibility of treated plants to *Pseudomonas syringae* pv. *DC3000 hrcC* suggesting a crucial role in energy supply during early stages of Pattern-Triggered immunity (PTI) establishment (Park et al., 2015).

Bearing in mind the priming potential of TCAs, and the positive link between BABA, plant defense responses and TCA components (Pastor et al., 2014; Thevenet et al., 2017), it was assessed here whether citrate and fumarate are also able to trigger the induction of endogenous BABA. The present data show that citrate treatment does not affect levels of BABA in the priming phase, therefore it can be hypothesized that the elevated TCA levels observed during BABA-induced priming are likely not responsible for the potentiation of a putative endogenous BABA pathway. As a consequence, TCAs seem to act as priming agent by a non-classical priming pathway independent of endogenous BABA.

**FIGURE 8:**
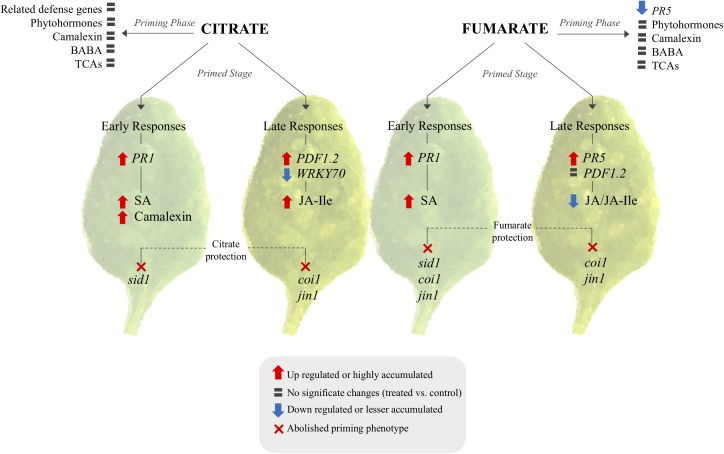
Signaling pathway proposed for citrate and fumarate induce priming in Arabidopsis against *Pst*DC3000. The scheme shows transcriptional and metabolomic changes induced by both TCAs during the priming phase and the primed stage (early and late responses of primed plants against bacterial infection) as well as phytohormone mutants in which citrate and fumarate were impaired in mounting priming. TCA-induced priming can be characterized in two branches: citrate and fumarate specific priming. In both cases the priming stage and priming phase exhibit distinct transcriptomic and metabolomics patterns. Citrate acts over *PR1*, SA and camalexin and is dependent on *sid1* for early defenses responses. Late defenses responses relay on JA pathway. Fumarate on the other hand relay in SA and JA signaling during early and late responses.

As a next step, an attempt was made to characterize the molecular and chemical mechanisms of TCA-mediated priming, in order to build a hypothetic signaling scheme (**Figure 8**). Generally, in Arabidopsis plants, altered levels of TCA cycle intermediates induce strong alterations in transcript abundances (Finkemeier et al., 2013), and transcriptional changes caused by TCAs are known to be specific for each metabolite (Müller et al., 2001). The behavior of specific transcript induction or repression by the intermediates of the TCA cycle confirm that these metabolites can act as a signaling molecules and strongly indicates a positive relationship between TCA components and plant defenses. Consequently, the question remains whether citrate and fumarate induce resistance by triggering the expression of defense genes only, or if they also prime plants for an augmented defense gene expression. Data shown here corroborate the fact that citrate and fumarate act as modifiers of transcriptional signaling during priming (**Figure 8**). Here, a group of phytohormone-related genes involved in Arabidopsis defenses was analyzed. The present findings indicate that citrate and fumarate act as priming inducers, since no significant transcriptional reprogramming occurs. Both chemical treatments are presumably triggering no additional costs to plants when no challenge or stress is present. Intriguingly, fumarate pre-treatment induces a different pattern of gene induction upon *Pst*DC3000 infection than citrate. Fumarate down-regulated *WRKY70* and *LOX2* gene expression at later time points after infection whereas *PR1* and *LOX2* were slightly induced at early time points upon *Pst*DC3000 infection. The distinct gene expression patterns observed in citrate- and fumarate-primed plants are in line with previous conclusions that alterations in specific TCA component levels result in unique transcriptome responses (Finkemeier et al., 2013). Especially, citrate pre-treatment primed for an enhanced *PR1* expression at early time points and a *PDF1.2* expression at later time points upon *Pst*DC3000 infection. This observation highlights the importance of the SA signaling pathway during priming and early defense responses (**Figure 8**), similarly as shown by Mhamdi et al. (2010). There, Arabidopsis mutants accumulating higher levels of citrate were more resistant to *Pst*DC3000 and showed a SA-regulated *PR* genes constitutively overexpressed.

In addition to hormone-related genes, actual levels of the phytohormones SA, JA and JA-Ile were quantified during the different priming phases induced by citrate and fumarate. As a general pattern, neither fumarate nor citrate induced phytohormone changes during the primed phase, as expected in light of the observed gene expression pattern (**Figure 8**). However, under *Pst*DC3000 infection, fumarate and citrate induced the accumulation of SA at early time point (24 hpi). Subsequently, JA and JA-Ile were down-regulated by fumarate at later time point whereas JA-Ile was upregulated by citrate. The hormone-dependent priming action of TCAs was further corroborated when monitoring TCA-induced priming in phytohormone mutants or Arabidopsis. For instance, the SA-impaired mutant *sid1* was insensitive to priming by citrate, and fumarate at early time point post infection, indicating an incomplete or inefficient priming mechanisms (**Figure 7**). Similarly, in the JA mutant *jin1*, the primed phenotype induced by citrate and fumarate was abolished in all of the analyzed time points. This mutant is known to constitutively express higher *PR1 levels* and to accumulate elevated levels of SA upon *Pst*DC3000 (Laurie-Berry et al., 2006). This suggests that in this specific genotype, priming by TCA does not exceed the already present constitutive resistance to bacteria by high endogenous levels of SA. Furthermore, the *coi1* mutant was able to mount a priming response by fumarate at 24 hpi, however, the phenotype was reversed at 72 hpi where elevated bacterial growth was observed compared to control plants. In the same genotype, citrate-induced priming was not effective anymore at 72 hpi, indicating that that JA signaling is crucial in mounting a citrate-triggered priming especially during the necrotrophic growth stage of *Pst*DC3000. Recently, JA treatment was demonstrated to have a protective effect against nematode infection in tomato plants, by upregulating amongst others organic acids like citrate and fumarate up to 19% (Bali et al., 2018). Despite the complexity of jasmonate-signaling pathways (Laurie-Berry et al., 2006), this new study reinforces the connection of citrate and JA pathway. However, the link between plant hormones and TCA-triggered priming is likely a multilayered one. For instance, fumarate treatment induced a depletion in JA/JA-Ile contents at later time points of *Pst*DC3000 infection, while *jin1* and *coi1* mutants were impaired to induce priming by this carboxylate. This suggests that not only the hormonal pathways are important during TCA-induced priming, but also the crosstalk between JA and SA. It is well known that JA extensively interacts with other plant hormone signaling pathways. Under *Pst*DC3000 infection, Arabidopsis plants produce both SA and jasmonates to modulate their defense responses (Block et al., 2005). This crosstalk is intriguingly complex and usually dose and time-specific (Kazan and Manners, 2008). Altogether, the data presented here suggest that predominantly SA rather than JA pathways are important for fumarate and citrate induced priming at early infection stages. Especially at later time points, JA seems to be required for a proper citrate-induced priming response (**Figure 8**). Notably, it has been recently demonstrated that plant defense induction by the avirulent *Pst* avrRpm1 strain leads to a reorganization of primary metabolism in systemic leaves characterized by a strong increment of fumarate and malate contents and a depletion of genes associated with JA and ethylene signaling (Schwachtje et al., 2018). Future work is required to better understand the connection between fumarate and JA signaling.

Another aspect of antibacterial defense is the induction of phyoalexins such as camalexin. Its accumulation is known to inhibit the growth of virulent *Pseudomonas syringae*. The natural variation of camalexin induction is well known to correlate with the resistance phenotype observed in a natural Arabidopsis population against *Pst*DC3000 (Zhang et al., 2014a). The data obtained here depict an induction of camalexin during *Pst*DC3000 infection development in Col-0 genotype as previously shown (Qiu et al., 2008). Importantly, citrate treatment greatly potentiated camalexin accumulation at early time point post infection, which was not observed in fumarate-induced priming. This finding follows along the general observation here that both TCAs prime plant defense on a distinct signaling pathway (**Figure 8**), which might depend on the status of both host and pathogen cells. This was confirmed when testing a generally necrotrophic pathogen. There, citrate but not fumarate was found to protect against *Plectosphaerella cucumerina BMM* (**Supplementary Figure S4**). The distinct mode of action of fumarate and citrate could be explained by their involvement in further plant defense strategies. For example, malate and fumarate (5–20 mM) treatments were previously demonstrated to reduce stomatal aperture in detached leaves of tomato plants after 2 h of incubation (Araújo et al., 2011). This might explain partially the resistance phenotype induced by fumarate in our study. While fumarate treatment was indeed able to induce resistance against *Pst*DC3000, the triggered molecular as well as the metabolic pattern was distinct from citrate. Further studies are required to decipher additional signaling cascades in TCA-induced priming against bacteria. These signaling cascades could involve additional defense layers such as pattern-triggered immunity (PTI) or effector-triggered immunity (ETI).

A plausible scenario of TCA-induced priming could not only involve the potentiation of defense layers, but also a positive feedback loop where TCA treatment itself upregulates intracellular levels of TCA flux. Data presented here demonstrate that endogenous TCA levels did not show any significant changes following treatment with citrate and fumarate, respectively at the time of inoculation with bacteria. This observation might suggest that at 48hpt both exogenously applied carboxylic acids had been catabolized by plants as expected due to the nature of both molecules as providers of carbon skeletons for a large range of biosynthetic process in plant tissues. Daloso et al. (2015) showed a rapid uptake and degradation of TCA intermediates by plants where redistribution of the isotope ^13^C after 15mM ^13^C-malate feed leaves were found in fumarate, succinate and malate intermediates already after 4 h of incubation. Even though intracellular levels of TCA were not significantly altered in the TCA-induced priming phase, it cannot be ruled out that citrate or fumarate exert a role as extracellular signaling molecule. For instance, citrate was previously shown to be the most abundant metabolite accumulated in the apoplast fluid after *Pseudomonas* infection; citrate levels increased from 300 μM to 1 mM at 4 hpi (O’Leary et al., 2016). Moreover, Anderson et al. (2014) demonstrated a dose-dependent signaling effect of citrate on *Pst*DC3000 gene expression. Apoplastic citrate levels at physiological concentrations of 50–200 μM strongly promoted the expression of the T3SS-secreted effector protein α-AvrPto but completely inhibited its expression at 1mM. This indicates an intriguing dose-dependent role of citrate as signal modulator of plant effector expression.

In summary, we show here that carboxylic acids can act as priming agents in Arabidopsis in response to *Pst*DC3000. They act by potentiating plant immunity, as no direct bacterial toxicity was observed. The capacity of these compounds to act as priming agents is supported by the finding that they do not induce defense genes during the priming phase, however, they potentiate defense gene expression in the primed state in a specific way. SA and JA pathways are eminent in TCA-induced priming, however, citrate and fumarate are likely acting over distinct signaling cascades. Further studies will help uncover the promising priming potential of TCAs, as well as TCA-mediated priming against different types of stress.

## Author Contributions

AB designed and executed the experiments, and wrote the manuscript. VP measured the TCA contents and helped to write the manuscript. GG quantified camalexin and supported for writing the manuscript. BM-M designed the experiments and wrote the manuscript.

## Conflict of Interest Statement

The authors declare that the research was conducted in the absence of any commercial or financial relationships that could be construed as a potential conflict of interest.

## Footnotes

1 www.sigmaaldrich.com

2 www.momentive.com

3 www.sigmaaldrich.com

4 http://www.bioline.com

5 www.graphpad.com
