# Supplementary material for: Tricarboxylates Induce Defense Priming Against Bacteria in Arabidopsis thaliana
Source: Front Plant Sci. 2018 Aug 20;9:1221. doi: 10.3389/fpls.2018.01221 (PMC6110165; doi:10.3389/fpls.2018.01221)
Supplement: Supplementary file 1 [file Presentation_1.PDF]

Supplementary Material

Tricarboxylates Induce Defense Priming Against Bacteria In *Arabidopsis thaliana*

Andrea Balmer [1], Victoria Pastor [2], Gaetan Glauser[3], Brigitte Mauch-Mani [1*]

*** Correspondence:**
Brigitte Mauch-Mani
brigitte.mauch@unine.ch

Supplementary Data

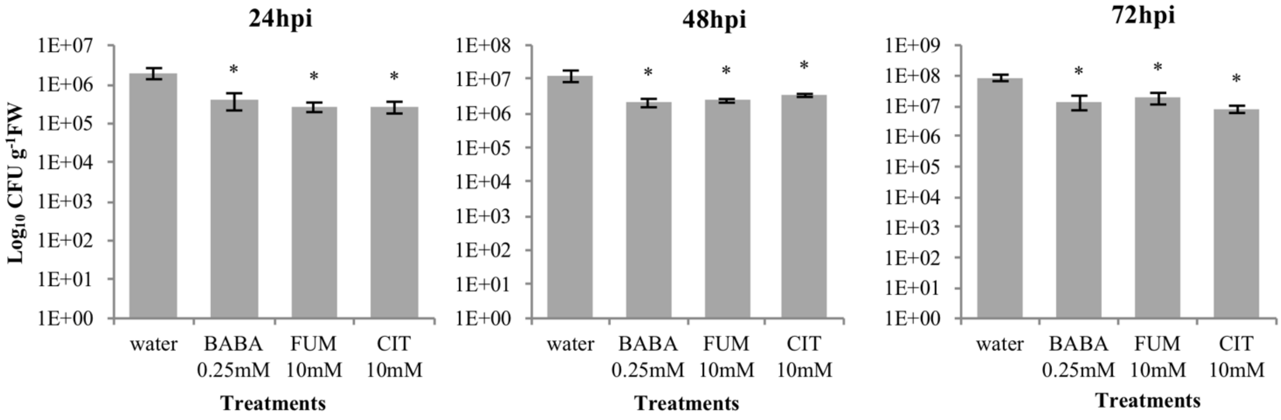

Supplementary Figure 1. In Arabidopsis, the TCAs citrate and fumarate confer resistance to *Pst*DC3000. Disease rate in colony forming units (*cfu)* per gram of fresh material at 24, 48 and 72hpi of Arabidopsis plants soil drenched with fumarate 10mM, citrate 10mM, water and BABA 0.25mM as a controls 2 days before dip-inoculation with *Pst*DC3000 (10^6cfu mL^{-1}). Data represent the mean ± SEM (n = 8 biological replicates). The experiments were repeated three times and a representative replicate is shown. Asterisks indicate significant differences: *P ≤0.05; (treatments vs. water) as determined by t-test.

.

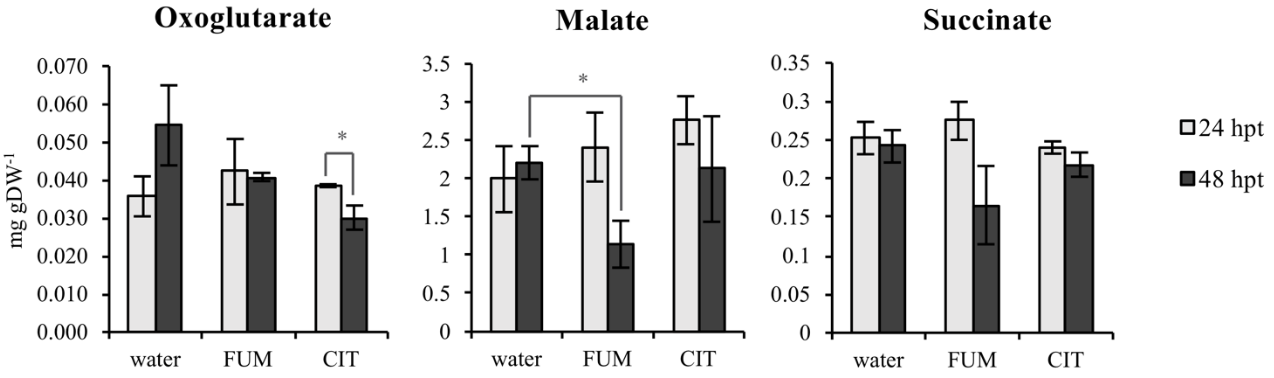

Supplementary Figure 2: Oxoglutarate, malate and succinate levels upon citrate and fumarate treatment in the priming phase (24hpt and 48hpt). Arabidopsis plants were collected 24 and 48h after soil drench with 10mM citrate and 10mM fumarate or water as a control. TCAs levels are expressed in mg gDW^{-1}. Data represent the mean ± SEM (*n* = 3 biological replicates) of one from three independent experiments. Asterisks indicate significant differences (treatments vs. control or 24hpt vs. 48hpt) as determined by *t*-test: $*P \leq 0.05$.

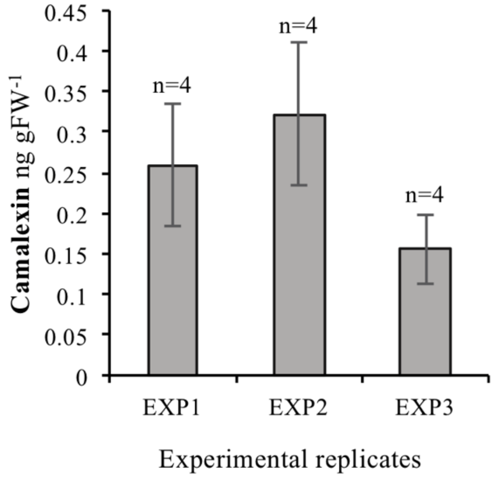

Supplementary Figure 3. Basal levels of camalexin in Arabidopsis plants (ng gFW^{-1}). Samples consist of four to five week-old Arabidopsis Col-0 leaves. Data represent the mean ± SE (*n* = 4 biological replicates). These experiments were repeated on three independent occasions (EXP1, EXP2, EXP3).

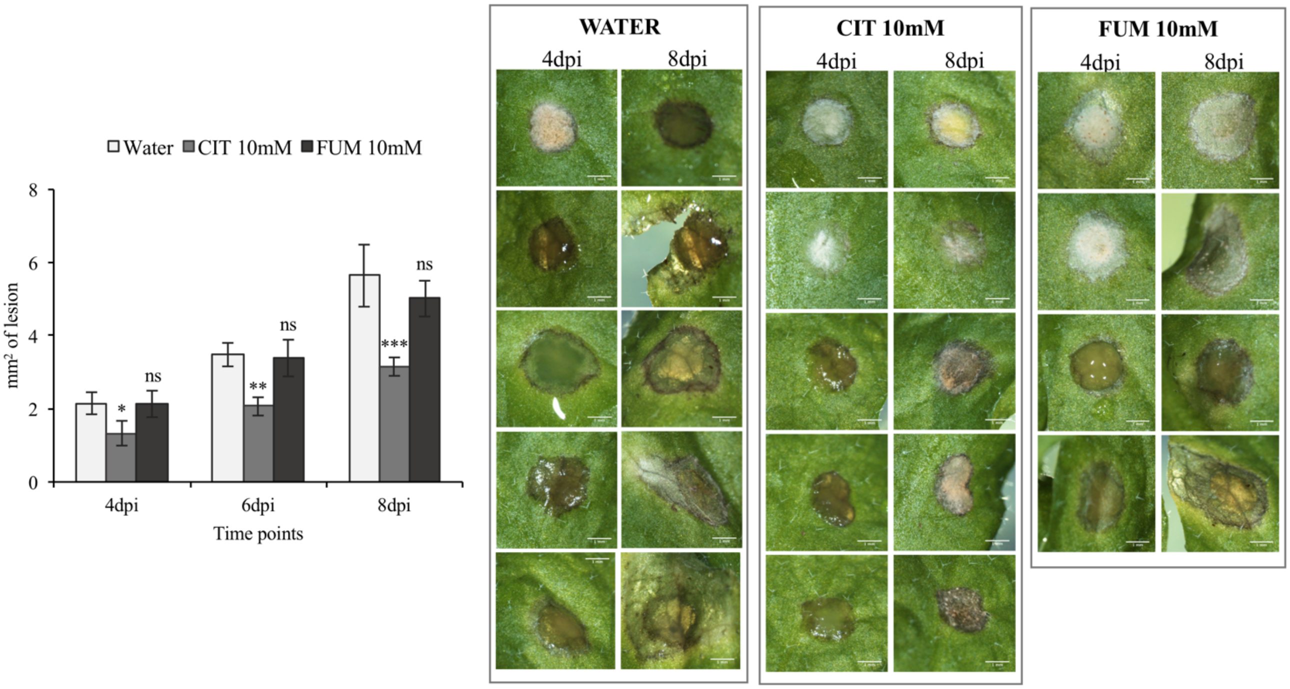

Supplementary Figure 4. Lesion diameter in mm^2 at 4dpi, 6dpi and 8dpi on Arabidopsis plants soil drenched with water, 10mM citrate and 10mM fumarate 2 days before drop inoculation with *Plectosphaerella cucumerina BMM* (5x10^6 spores mL^{-1}). Photos from leaves were taken 4dpi and 8pi. Data represent the mean ± SE (*n* = 24 biological replicates). Asterisks indicate significant differences: $*P \leq 0.05$; $** P \leq 0.01$; $*** P \leq 0.001$ (treatments vs. water) as determined by *t*-test.

Name	Sequence	Efficiency
AtPR5_fw	GAC TGT GGC GGT CTA AGA TGT	0.99
AtPR5_rev	TGA ATT CAG CCA GAG TGA CG	0.99
AtWRKY70_fw	GGG TTC TCA GGA TCT CAT GG	0.99
AtWRKY70_rev	TCA TTT TCG CTA AAC TCG AAA TC	0.99
AtPDF1.2_fw	GCA ATG GTG GAA GCA CAG A	0.97
AtPDF1.2_rev	GCA AAC CCC TGA CCA TGT	0.97
AtPR1_fw	TGA TCC TCG TGG GAA TTA TGT	0.99
AtPR1_rev	TGC ATG ATC ACA TCA TTA CTT CAT	0.99
AtLOX2_fw	CTT ACC CGC GGA TCT CAT C	0.97
AtLOX2_rev	ACT CCA TGT TCT GCG GTC TT	0.97
At_ACTIN_fw	TGG GAT GAA CCA GAA GGA TG	0.99
At_ACTIN_rev	AAG AAT ACC TCT CTT GGA TTG TGC	0.99
At_PAD3_fw	ACA AGA ACA GGG CAA GGA AA	0.99
At_PAD3_rev	GAT CAG GGG TAA GAG GAC GA	0.99
At_SAND_fw	TGG TAG CCA CAC CGA ATT TA	0.9
At_SAND_rev	GTT AGC CGA TGC AAC CTC AT	0.9

Supplementary Table 1: Primer sequences and primer efficiency calculated with the help of a dilution curve.
